# Novel duplex TaqMan-based quantitative PCR for rapid and accurate diagnosis of *Leishmania* (*Mundinia*) *martiniquensis* and *Leishmania* (*Mundinia*) *orientalis*, responsible for autochthonous leishmaniasis in Thailand

**DOI:** 10.1016/j.crpvbd.2024.100217

**Published:** 2024-09-24

**Authors:** Kanok Preativatanyou, Nopporn Songumpai, Pathamet Khositharattanakool, Rinnara Ampol, Chulaluk Promrangsee, Chatchapon Sricharoensuk, Kobpat Phadungsaksawasdi, Thanapat Pataradool, Tomas Becvar, Barbora Vojtkova, Petr Volf, Padet Siriyasatien

**Affiliations:** a Center of Excellence in Vector Biology and Vector-Borne Disease, Chulalongkorn University, Thailand; b Department of Parasitology, Faculty of Medicine, Chulalongkorn University, Bangkok, Thailand; c Division of Infectious Diseases, Department of Internal Medicine, Hatyai Hospital, Songkhla, Thailand; d School of Medicine, Mae Fah Luang University, Chiang Rai, Thailand; e Biomedical Technology Research Group for Vulnerable Populations, Mae Fah Luang University, Chiang Rai, Thailand; f Interdisciplinary Program of Biomedical Sciences, Graduate School, Chulalongkorn University, Bangkok, Thailand; g Department of Parasitology, Faculty of Science, Charles University, Prague, Czech Republic

**Keywords:** *Leishmania martiniquensis*, *Leishmania orientalis*, *Mundinia*, Duplex qPCR, ITS1, *HSP70-I* intergenic region

## Abstract

The World Health Organization has recently declared Thailand a leishmaniasis hotspot in Southeast Asia due to the continuous increase in new symptomatic and asymptomatic cases over the years. This emerging parasitic disease is known to be caused by two autochthonous species of *Leishmania* belonging to the newly described subgenus *Mundinia*, namely *L. martiniquensis* and *L. orientalis*. In Thailand, clinical cases due to *L. martiniquensis* typically present with visceral leishmaniasis, whereas *L. orientalis* mainly causes localized cutaneous leishmaniasis. Although *Leishmania* species confirmation is essential for clinical diagnosis and treatment planning, the availability of highly accurate and rapid diagnostic methods remains limited. In this study, we developed a duplex TaqMan quantitative PCR assay using newly designed species-specific primers and probes based on sequences from the nucleotide and genome databases of *Leishmania* spp. retrieved from GenBank. The duplex qPCR assay was optimized to specifically amplify the internal transcribed spacer 1 (ITS1) of *L. martiniquensis* and the heat shock protein 70 (type I) intergenic region (*HSP70-I* IR) of *L. orientalis* with high amplification efficiencies. The performance of the optimized duplex qPCR was evaluated by analyzing 46 DNA samples obtained from cultures, and clinical and insect specimens, consistent with the results of the previously validated 18S rRNA-qPCR and ITS1-PCR. The duplex qPCR could detect both species of *Leishmania* at a limit of detection of one copy per reaction and did not cross-amplify with other pathogen DNA samples. Standard curves of the singleplex and duplex assays showed good linearity with excellent amplification efficiency. Using conventional ITS1-PCR and plasmid sequencing as a reference standard assay, the duplex qPCR showed diagnostic sensitivity and specificity of 100% and positive and negative predictive values of 100% for both *Leishmania* species with a perfect level of agreement (kappa = 1.0). The novel duplex TaqMan-based qPCR has shown to be a rapid, cost-effective, and highly accurate diagnostic tool for the simultaneous detection and identification of two autochthonous *Leishmania* spp. in a variety of clinical and entomological samples. This will greatly facilitate early diagnosis, treatment monitoring, and surveillance, especially in leishmaniasis-endemic areas where sequencing-based diagnosis is not routinely available.

## Highlights

• A novel duplex qPCR assay was developed to detect *Leishmania martiniquensis* and *L. orientalis* simultaneously.
• Primers and TaqMan probes were designed to detect *L. martiniquensis* ITS1 and *L. orientalis HSP70-I* intergenic region.
• Duplex qPCR was optimized, showing high analytical sensitivity and specificity, and excellent amplification efficiency.
• Duplex qPCR was tested with a variety of biological samples, showing diagnostic sensitivity and specificity of 100%.
• This novel assay can be a valuable diagnostic tool, especially in endemic areas where DNA sequencing is not routine.

## Graphical abstract

**Figure:**
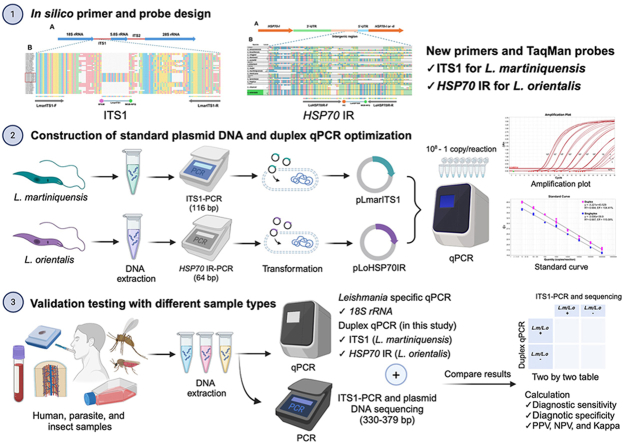

## 1 Introduction

Leishmaniasis is a neglected vector-borne, potentially life-threatening disease caused by an obligate intracellular parasite belonging to the genus *Leishmania* (Burza et al., 2018). This disease has been known to be transmitted by the bites of phlebotomine sand flies and is widespread in tropical and subtropical countries worldwide (WHO, 2023). According to recent epidemiological data from the World Health Organization, an estimated 600,000 to 1 million new cases of cutaneous leishmaniasis and 50,000–90,000 new cases of visceral leishmaniasis occur each year worldwide (WHO, 2023). More than 50 species of *Leishmania* have been described, of which more than 20 can infect humans (Akhoundi et al., 2016). The main clinical spectrum of the disease includes cutaneous, mucocutaneous, and visceral leishmaniasis, depending on the infecting species and the host immune status (Aronson et al., 2016; Mann et al., 2021). Species of *Leishmania* are taxonomically divided into four subgenera, including *Leishmania*, *Viannia*, *Sauroleishmania*, and the newly described *Mundinia* (Espinosa et al., 2018). The new subgenus *Mundinia* consists of six member species, namely *L. enriettii* (Muniz and Medina, 1948), *L. martiniquensis* (Pothirat et al., 2014), *L. orientalis* (Jariyapan et al., 2018), *L. chancei* (Kwakye-Nuako et al., 2015, 2023), *L. procaviensis* (Kwakye-Nuako et al., 2023), and *L. macropodum* (Dougall et al., 2011; Rose et al., 2004).

Currently, leishmaniasis is considered an important public health problem in Southeast Asia, with Thailand being an endemic hotspot due to the increasing number of new clinical cases and asymptomatic individuals, particularly in the northern and southern provinces of the country (Leelayoova et al., 2017; Sarasombath, 2018; Srivarasat et al., 2022; Preativatanyou et al., 2023; WHO, 2023). This emerging disease is caused by two autochthonous *Leishmania* spp. of the new subgenus *Mundinia*, namely *L. martiniquensis* (Pothirat et al., 2014; Songumpai et al., 2022; Srivarasat et al., 2022; Preativatanyou et al., 2023) and *L. orientalis* (Jariyapan et al., 2018; Anugulruengkitt et al., 2022), previously named *Leishmania* sp. ‘siamensis’ lineages PG and TR, respectively (Mungthin et al., 2021). Most of the autochthonous cases in Thailand have been diagnosed as infections with *L. martiniquensis*, which typically causes visceral leishmaniasis, with the potential for concomitant cutaneous and mucocutaneous leishmaniasis, particularly in immunosuppressed patients (Srivarasat et al., 2022). In contrast, sporadic cases of infection with *L. orientalis* with a typical presentation of localized cutaneous leishmaniasis have been reported (Jariyapan et al., 2018; Anugulruengkitt et al., 2022). However, the biology, transmission, and epidemiology of these two common *Leishmania* spp. in human and animal reservoirs, especially in the endemic areas, remain poorly understood due to limited diagnostic capabilities.

Due to the wide range of clinical manifestations and the diversity of parasite species, leishmaniasis is often difficult to diagnose. Traditionally, suspected cases of leishmaniasis can be diagnosed by direct microscopy, histology, parasite culture, and serology (Aronson et al., 2016; Mann et al., 2021). Nevertheless, these conventional detection methods cannot identify the parasite species due to the morphological similarity between parasite species. In recent years, molecular diagnostics, particularly polymerase chain reaction (PCR)-based assays, have been developed as highly accurate diagnostic tools for several infectious diseases, including leishmaniasis. However, conventional PCR typically requires DNA sequencing for species identification or confirmation. In addition, species identification for mixed infection samples cannot be based on direct Sanger sequencing, which provides a single sequence chromatogram representing only a single species (Ohta et al., 2023; Ampol et al., 2024). This limitation could be overcome by cloning the PCR product into the plasmid vector and collecting multiple recombinant clones for Sanger sequencing. However, this procedure is time-consuming and requires specific equipment, making it unsuitable for rapid diagnosis, particularly in leishmaniasis-endemic areas where sequencing-based diagnostic methods are not commonly accessible.

Quantitative PCR (qPCR) using fluorescent-labeled, target-specific probes provides an alternative means of detection and identification with high sensitivity and accuracy (Arya et al., 2005; Kralik and Ricchi, 2017). Primers and probes can be designed to be species-specific, and the fluorescent signal will be emitted only when they hybridize with the DNA of the target species, allowing detection during amplification. Importantly, probe-based qPCR is also less time-consuming and does not require post-PCR processing steps which can cause the risk of contamination. More importantly, this molecular technique has been successfully developed to simultaneously detect and identify multiple pathogen species in a single reaction without the necessity of sequencing confirmation. Several probe-based qPCR methods have been developed for the detection and identification of other *Leishmania* spp. (Galluzzi et al., 2018). Most common targets previously described for detection and species differentiation include non-protein coding regions such as kinetoplast DNA minicircles and ribosomal RNA genes (rDNA), as well as protein-coding sequences such as heat shock protein 70 kDa (*HSP70*), glucose-6-phosphate dehydrogenase, and DNA polymerase (Galluzzi et al., 2018). Accordingly, we speculated that duplex probe-based qPCR will be beneficial for the clinical diagnosis and epidemiological surveillance of autochthonous leishmaniasis caused by these two *Leishmania* (*Mundinia*) species. In addition, the probe-based qPCR technique has never been explored for development as a diagnostic platform for leishmaniasis in Thailand.

Therefore, we aimed to develop and validate a rapid and highly sensitive probe-based duplex qPCR assay for the simultaneous detection and identification of two common autochthonous *Leishmania* species, *L. martiniquensis* and *L. orientalis*. The primers and probes were designed to detect these two *Leishmania* species based on sequence analysis of the internal transcribed spacer 1 (ITS1) and the intergenic region of heat shock protein 70 (type I) gene (*HSP70-I* IR) retrieved from the GenBank database. The diagnostic performance of the duplex qPCR assay was evaluated by analyzing several biological samples in comparison with the previously developed methods, including conventional ITS1-PCR and 18S rRNA-qPCR. The novel duplex qPCR assay developed in this study will greatly facilitate clinicians in the rapid and accurate diagnosis of leishmaniasis, confirmation of suspected co-infection by these two species of *Leishmania*, treatment monitoring, and epidemiological surveillance of this neglected disease, especially in leishmaniasis-endemic areas of Thailand.

## 2 Materials and methods

### 2.1 Specimen collection and DNA extraction

To evaluate the performance of the *L.martiniquensis*/*L. orientalis* duplex qPCR assay, a total of 46 DNA samples from humans, parasite cultures, and insects were tested. The sample collection consisted of 19 samples from 13 patients previously diagnosed with leishmaniasis; 4 samples from 4 uninfected healthy individuals; 4 samples from 4 patients infected with *Plasmodium falciparum*, *P. vivax*, *P. knowlesi*, and *Histoplasma capsulatum*; 12 samples of *Leishmania* spp. promastigote cultures; 2 samples of *Trypanosoma* sp. and *Crithidia* sp. cultures; and 5 samples of *Culicoides* biting midges collected from the house of the leishmaniasis patient in Songkhla Province, Southern Thailand.

For formalin-fixed, paraffin-embedded (FFPE) clinical samples, DNA was extracted using the QIAamp DNA FFPE Tissue Kit (Qiagen, Hilden, Germany). For other sample types, DNA was extracted using the DNeasy Blood & Tissue Kit (Qiagen, Hilden, Germany). The concentration and purity of the extracted DNA were assessed using a NanoDrop 2000c spectrophotometer (Thermo Fisher Scientific, Waltham, MA, USA), and preserved at −20 °C until further analysis.

### 2.2 18S rRNA-qPCR

Forty-six previously extracted genomic DNA (gDNA) samples were initially screened for *Leishmania* spp. by qPCR specific for the conserved region of the 18S ribosomal RNA (18S rRNA) gene, as previously described (van der Meide et al., 2008). The qPCR reactions were performed using the QuantStudio™ 5 Real-Time PCR System (Applied Biosystems, Foster City, CA, USA) in a total volume of 20 μl containing 10 μl of TaqMan™ Fast Advanced Master Mix (Thermo Scientific, Waltham, MA, USA), 1 μl of each 10 μM primer (Le18S-F and Le18S-R), 0.5 μl of 10 μM Le18S probe, 2 μl of gDNA, and 5.5 μl of nuclease-free water. Thermal conditions included initial denaturation at 95 °C for 2 min; 45 cycles of 95 °C for 15 s and 60 °C for 15 s. A Ct-value < 40 was considered positive. The internal control for DNA integrity and the presence of amplification inhibitors in FFPE-derived DNA samples was verified by the detection of a fragment of the human ribonuclease P gene as described elsewhere (Tatti et al., 2011).

### 2.3 Conventional ITS1-PCR, cloning, and Sanger sequencing

All 46 gDNA samples were further analyzed by conventional PCR targeting the internal transcribed spacer 1 region (ITS1) region of *Leishmania* species. A set of *Leishmania*-specific LeF and LeR primers, as listed in Table 1, was used to amplify an amplicon product of approximately 330–379 bp, encompassing the full-length ITS1 and its flanking partial 18S rRNA and 5.8S rRNA regions (Spanakos et al., 2008). ITS1-PCR reactions were performed in a 20 μl mixture containing 2 μl of gDNA, 10 μl of 2× KAPA HiFi HotStart ReadyMix (Roche, Basel, Switzerland), 0.8 μl of each 10 μM primer, and 6.4 μl of nuclease-free water. The amplification program included initial denaturation at 95 °C for 5 min, followed by 40 cycles of 98 °C for 30 s, 65 °C for 30 s, and 72 °C for 1 min, and final extension at 72 °C for 5 min. Amplicons were verified by 1.5% (*w*/*v*) agarose gel electrophoresis stained with RedSafe™ nucleic acid staining solution (iNtRON Biotechnology Inc., Seongnam, Korea), and visualized using the GelDoc Go Imaging System (Bio-Rad, Hercules, CA, USA).

**Table 1:** Oligonucleotide primers and fluorescent probes for the detection of *Leishmania* spp. in this study.

Species/Target	Primer and probe	Oligonucleotide sequence (5′→3′)	Amplicon size (bp)	Reference
***Leishmania* spp.**				
ITS1 (PCR)	LeF	TCCGCCCGAAAGTTCACCGATA	330–379	Spanakos et al. (2008)
	LeR	CCAAGTCATCCATCGCGACACG		
18S rRNA (qPCR)	Le18S-F	CCAAAGTGTGGAGATCGAAG	171	van der Meide et al. (2008)
	Le18S-R	GGCCGGTAAAGGCCGAATAG		
	Le18S probe	6FAM-ACCATTGTAGTCCACACTGC-MGB-NFQ		
***L. martiniquensis***				
ITS1 (qPCR)	LmarITS1-F	GCAGCTGGATCATTTTCCGA	116	This study
	LmarITS1-R	TGTTTGTGTATGTGGGAAAGGC		
	LmarITS1 probe	6FAM-AGGTAGAGAGTAGTAGAATAC-MGB-NFQ		
***L. orientalis***				
*HSP70-I* IR (qPCR)	LoHSP70IR-F	AAGCATACGCCTCTCTCTCTATCC	64	This study
	LoHSP70IR-R	GAAGGAGACGYTCCACAGACA		
	LoHSP70IR probe	VIC-CTCAGCTCTCCTGGAGC-MGB-NFQ		

PCR amplicons obtained from the positive samples were purified using the QIAquick PCR Purification Kit (Qiagen, Hilden, Germany) and cloned into pGEM® T-Easy plasmids using the LigaFast™ Rapid DNA Ligation System (Promega Corporation, Madison, WI, USA). The ligations were chemically transformed into *Escherichia coli* DH5α competent cells and plated on the Luria-Bertani (LB) agar plate supplemented with ampicillin, X-Gal, and IPTG for blue/white colony screening. Positive colonies with inserted plasmids were white and chosen for further inoculation. Five colonies from each positive sample were inoculated into LB broth supplemented with ampicillin and incubated at 37 °C. Plasmids containing the insert were extracted using the Invisorb® Spin Plasmid Mini Kit (STRATEC, Birkenfeld, Germany) and subjected to Sanger DNA sequencing using the T7 promoter sequencing primer. The species of each positive sample was identified by alignment of the ITS1 sequences obtained against the GenBank reference using the Basic Local Alignment Search Tool (https://blast.ncbi.nlm.nih.gov/Blast.cgi).

### 2.4 *In silico* design of primers and probes for *L. martiniquensis*/*L. orientalis* duplex qPCR

**Fig. 1:**
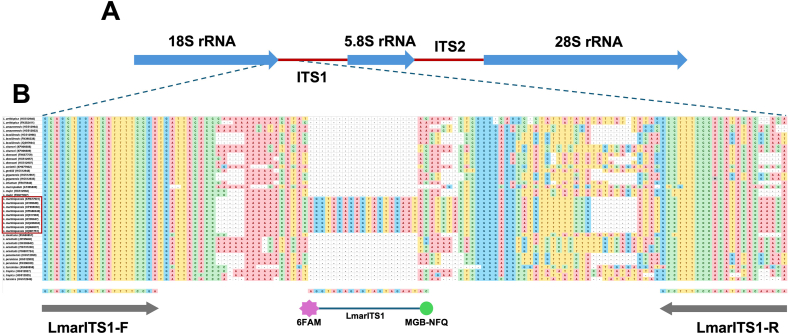
**A** Schematic representation of the rRNA transcription unit encoding 18S rRNA, ITS1, 5.8S rRNA, ITS2, and 28S rRNA in *Leishmania* species. **B** ClustalW alignment of the 5′-end of the ITS1 region shows two highly conserved sequence blocks used to design forward and reverse primers for amplification of *L. martiniquensis*, generating an amplicon of 116 bp. The LmarITS1 probe was then designed based on the sequence found exclusively in *L. martiniquensis*.

**Fig. 2:**
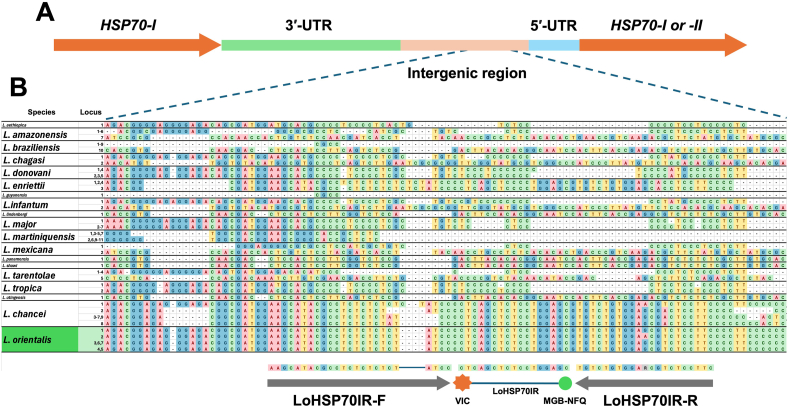
**A** Schematic representation of the intergenic region between two *Leishmania HSP70-I* loci. **B** ClustalW alignment of the *HSP70-I* IR revealed the sequence region used to design primers and a probe for amplification of *L. orientalis* with a product size of 64 bp. Note that the closely related *L. chancei* could potentially be amplified using primers and a probe designed for *L. orientalis* in this study.

The ITS1 and the *HSP70-I* IR sequences of *Leishmania* spp. downloaded from the GenBank database were used as specific targets for the development of the *L. martiniquensis*/*L. orientalis* duplex qPCR assay, as detailed in Supplementary Tables S1 and S2. Representative sequences of each locus from *Leishmania* spp. were aligned using ClustalW implemented in BioEdit version 7.2.6 (Hall, 1999) to reveal the regions of high intraspecific conservation, which allowed us to design custom primers and probes for the duplex qPCR assay, as illustrated in Fig. 1, Fig. 2. Primer Express™ software version 3.0.1 (Applied Biosystems, Foster City, CA, USA) was used to calculate melting temperatures and avoid potential primer dimer formation. Two sets of primers (LmarITS1-F/LmarITS1-R and LoHSP70IR-F/LoHSP70IR-R) were newly designed in the present study to amplify ITS1 and *HSP70-I* IR with products of approximately 116 bp and 64 bp, respectively. The TaqMan probes specific for *L. martiniquensis* and *L. orientalis* were LmarITS1 and LoHSP70IR with the 5′-end labeled with fluorochromes 6-carboxyfluorescein (6FAM) and 2′-chloro-7′-phenyl-1,4-dichloro-6-carboxyfluorescein (VIC), respectively, and the 3′-end labeled with a minor groove binder-nonfluorescent quencher (MGB-NFQ) (Applied Biosystems, Foster City, CA, USA). Table 1 provides information on the newly designed primers and probes used in this study.

### 2.5 Construction of standard plasmid DNA

Standard plasmids were constructed by cloning the target fragment amplified from promastigote cultures using the same primer set designed for the duplex qPCR assay as shown in Table 1. Genomic DNA samples extracted from *L. martiniquensis* MHOM/TH/2012/CULE1 and *L. orientalis* MHOM/TH/2021/CULE5 were used as templates for PCR amplification. The PCR components were set up in a volume of 20 μl containing 2 μl of reference gDNA, 10 μl of 2× KAPA HiFi HotStart ReadyMix (Roche, Basel, Switzerland), 0.8 μl of each 10 μM primer, and 6.4 μl of nuclease-free water. Amplification cycling conditions included initial denaturation at 95 °C for 5 min, followed by 35 cycles of 98 °C for 30 s, 57 °C (LmarITS1-F/LmarITS1-R) for 30 s and 60 °C (LoHSP70IR-F/LoHSP70IR-R) for 30 s, and 72 °C for 30 s, and final extension at 72 °C for 5 min. Amplicons were verified using 2% (*w*/*v*) agarose gel electrophoresis with staining and visualization as previously described. Both *L.* *martiniquensis* ITS1 and *L. orientalis HSP70-I* IR fragments were cleaned up using ExoSAP-IT reagent (Thermo Scientific, Waltham, MA, USA) and then cloned into the pGEM® T-Easy vector as previously described. The presence of the insert was confirmed by Sanger sequencing. Standard plasmids (pLmarITS1 and pLoHSP70IR) were extracted from the positive clones and quantified on a Qubit™ 4 fluorometer using the Qubit™ dsDNA High Sensitivity Assay Kit (Thermo Scientific, Waltham, MA, USA).

### 2.6 Duplex qPCR optimization

The optimization of the duplex qPCR assay was performed by varying the concentrations of primers and probes specific for *L. martiniquensis* and *L. orientalis*. The duplex qPCR reactions were determined using the following thermocycling conditions: 95 °C for 2 min followed by 45 cycles of 95 °C for 15 s and 60 °C for 15 s. The duplex qPCR reactions were performed in a 20 μl mixture containing 10 μl of TaqMan™ Fast Advanced Master Mix (Thermo Scientific, Waltham, MA, USA), 1 ng of each standard plasmid (pLmarITS1 and pLoHSP70IR), primers at varying concentrations (0.2–0.8 μM) and probes at varying concentrations (0.1–0.5 μM) and nuclease-free water. The probe concentration was maintained at 0.3 μM to optimize the primer concentration. Similarly, the primer concentration was fixed at 0.5 μM to optimize the probe concentration. The 6FAM and VIC fluorophore channels were chosen to measure the fluorescence signal emitted by each reporter dye at each PCR cycle for subsequent determination of the threshold cycle (Ct).

### 2.7 Analysis of PCR efficiency, sensitivity, and specificity

The standard plasmids were used to construct a standard curve. The plasmid copy number was calculated from the size and concentration of the standard plasmid DNA using the DNA copy number calculator in Thermo Scientific Web Tools (https://www.thermofisher.com). The standard plasmid template of each species was prepared in a 10-fold serial dilution, ranging from 10^8^ copies to 1 target copy per reaction. The duplex qPCR assay was performed separately for each species using serially diluted plasmids with optimized primer and probe concentrations, and all reactions were spiked with 10^5^ copies of the standard plasmid of the counterpart species. The singleplex assay for each species was also performed using the same optimized conditions. Ct-values at each dilution were determined in triplicate and plotted against the logarithm of the initial template amount. The correlation coefficient (*R*^2^) and the slope of each standard curve plot were calculated. The amplification efficiency (*E*) for both the singleplex and duplex assays was determined from the dilution factor and the slope of each standard curve using the following equation: *E* = (−1 + 10^(−1/slope)^) × 100 (Ruijter et al., 2021).

In this study, the sensitivity and specificity of the assay were determined at both analytical and diagnostic levels. Analytical sensitivity was determined based on the limit of detection (LOD), which is the lowest amount of target per reaction that can be reliably detected. Analytical specificity was assessed using the duplex qPCR results of the samples devoid of *L. martiniquensis* and *L. orientalis* DNA, including other *Leishmania* spp., *Crithidia* sp., *Trypanosoma* sp., *Plasmodium falciparum*, *P. vivax*, *P. knowlesi*, *Histoplasma capsulatum*, and *Homo sapiens*.

Diagnostic sensitivity and specificity were also evaluated by comparing the results of the duplex qPCR with those of ITS1-PCR and plasmid sequencing as a reference standard for *Leishmania* spp. detection and identification in a two-by-two table. Diagnostic sensitivity for each species was calculated as the number of true positive samples correctly diagnosed by both assays divided by the total number of true positive and false negative samples. Diagnostic specificity for each species was calculated as the number of true negative samples correctly diagnosed by both assays divided by the total number of true negative and false positive samples. A positive predictive value (PPV), or precision, was calculated as the number of true positive samples divided by the total number of true positive and false positive samples. A negative predictive value (NPV) was calculated as the number of true negative samples divided by the total number of true negative and false negative samples (Šimundić, 2009). Additionally, the level of agreement between the duplex qPCR and the reference assay for each species was determined using Cohen's kappa statistic value (Cohen, 1960). Kappa values were interpreted as the degree of agreement as formerly described (Landis and Koch, 1977).

## 3 Results

### 3.1 Detection of *Leishmania* species by 18S rRNA-qPCR and ITS1-PCR coupled with plasmid sequencing

A total of 46 extracted DNA samples were preliminarily screened for *Leishmania* DNA using 18S rRNA-qPCR. As shown in Table 2, 18S rRNA-qPCR was positive in 37 DNA samples, including 19 from all leishmaniasis patients, 12 from *Leishmania* spp. cultures, 1 from *Crithidia* culture, and 5 from *Culicoides* midge samples. Ct-values of the qPCR assay ranged from 13.38 to 37.87. Conventional ITS1-PCR followed by cloning and plasmid sequencing was also performed to detect and identify *Leishmania* spp. in all positive samples. Most of the PCR results were in concordance with the qPCR results. Thirty-six samples tested positive except for one sample from a *Crithidia* culture, which was not detected by ITS1-PCR. Sequence analysis of 19 clinical samples from leishmaniasis patients revealed single infections with *L. martiniquensis*, *L. orientalis*, and *L. major* in 15, 1, and 3 samples, respectively. The species of all 12 *Leishmania* spp. cultures were consistently confirmed. Among the *Culicoides* samples, all 5 samples were infected with *L. martiniquensis* and 3 of them were co-infected with *L. orientalis*.

**Table 2:** Summary of *Leishmania* detection in clinical, culture, and insect specimens using 18S rRNA-qPCR, *L. martiniquensis*/*L. orientalis* duplex qPCR, and conventional ITS1-PCR followed by plasmid DNA sequencing.

Patient ID	Sample ID	Sample information		*Leishmania* 18S rRNA qPCR Ct-value (6FAM)	Duplex qPCR		ITS1-PCR and plasmid DNA sequencing
		Type of original sample	Tissue or parasite		*L. martiniquensis* Ct-value (6FAM)	*L. orientalis* Ct-value (VIC)	
1	1	FFPE	Human skin nodule	22.34	22.12	Negative	*L. martiniquensis*
2	2	FFPE	Human skin nodule	27.86	27.67	Negative	*L. martiniquensis*
3	3	FFPE	Human skin nodule	26.56	25.34	Negative	*L. martiniquensis*
	4	FFPE	Human bone marrow	23.98	22.97	Negative	*L. martiniquensis*
	5	FFPE	Human bone marrow	23.47	22.49	Negative	*L. martiniquensis*
4	6	FFPE	Human skin nodule	25.45	25.12	Negative	*L. martiniquensis*
5	7	FFPE	Human skin nodule	27.85	Negative	29.32	*L. orientalis*
6	8	FFPE	Human bone marrow	33.65	32.33	Negative	*L. martiniquensis*
7	9	Saliva	Human saliva	30.87	31.52	Negative	*L. martiniquensis*
	10	Fresh tissue	Human bone marrow	24.53	24.14	Negative	*L. martiniquensis*
8	11	FFPE	Human bone marrow	32.12	31.49	Negative	*L. martiniquensis*
9	12	Blood	Human blood	35.37	35.44	Negative	*L. martiniquensis*
10	13	Fresh tissue	Human skin nodule	13.38	13.65	Negative	*L. martiniquensis*
	14	Saliva	Human saliva	32.47	31.74	Negative	*L. martiniquensis*
	15	Blood	Human blood	32.51	31.95	Negative	*L. martiniquensis*
	16	Body fluid	Human ascitic fluid	29.25	28.69	Negative	*L. martiniquensis*
11	17	Swab	Human skin ulcer	24.13	Negative	Negative	*L. major*
12	18	Saliva	Human saliva	31.19	Negative	Negative	*L. major*
13	19	Saliva	Human saliva	35.10	Negative	Negative	*L. major*
NA	20–23	Blood	Negative human blood	Negative	Negative	Negative	Negative
NA	24	Blood	*P. falciparum*	Negative	Negative	Negative	Negative
NA	25	Blood	*P. vivax*	Negative	Negative	Negative	Negative
NA	26	Blood	*P. knowlesi*	Negative	Negative	Negative	Negative
NA	27	FFPE	*H. capsulatum*	Negative	Negative	Negative	Negative
NA	28	Culture	*L. martiniquensis* CULE1	23.24	22.87	Negative	*L. martiniquensis*
NA	29	Culture	*L. martiniquensis* CULE4	24.84	23.96	Negative	*L. martiniquensis*
NA	30	Culture	*L. martiniquensis* CULE6	22.56	23.18	Negative	*L. martiniquensis*
NA	31	Culture	*L. martiniquensis* CULE7.1	24.36	23.89	Negative	*L. martiniquensis*
NA	32	Culture	*L, martiniquensis* CULE8	23.47	23.84	Negative	*L. martiniquensis*
NA	33	Culture	*L. orientalis* CULE5	23.37	Negative	25.46	*L. orientalis*
NA	34	Culture	*L. macropodum*	25.32	Negative	Negative	*L. macropodum*
NA	35	Culture	*L. mexicana*	23.12	Negative	Negative	*L. mexicana*
NA	36	Culture	*L. braziliensis*	22.23	Negative	Negative	*L. braziliensis*
NA	37	Culture	*L. infantum*	24.63	Negative	Negative	*L. infantum*
NA	38	Culture	*L. major*	26.32	Negative	Negative	*L. major*
NA	39	Culture	*L. tarentolae*	25.64	Negative	Negative	*L. tarentolae*
NA	40	Culture	*Trypanosoma* sp.	Negative	Negative	Negative	Negative
NA	41	Culture	*Crithidia* sp.	37.87	Negative	Negative	Negative
NA	42	Insect	*C. insignipennis*	35.26	34.94	Negative	*L. martiniquensis*
NA	43	Insect	*C. sumatrae*	32.14	31.58	Negative	*L. martiniquensis*
NA	44	Insect	*C. fulvus*	33.26	31.89	34.90	*L. martiniquensis* and *L. orientalis*
NA	45	Insect	*C. shortti*	33.98	30.81	37.78	*L. martiniquensis* and *L. orientalis*
NA	46	Insect	*C. sumatrae*	34.26	30.19	38.17	*L. martiniquensis* and *L. orientalis*

*Abbreviations*: FFPE, formalin-fixed, paraffin-embedded sample; NA, not applicable; C., *Culicoides*; H., *Histoplasma*; P., *Plasmodium*; L., *Leishmania*.

### 3.2 Standardization of duplex qPCR

**Fig. 3:**
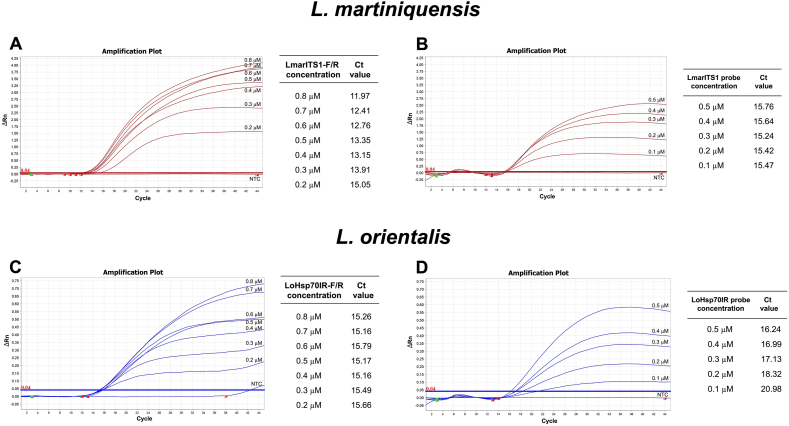
Optimization of primer and probe concentrations for the duplex qPCR assay using 1 ng/μl of pLmarITS1 and pLoHSP70IR standard plasmids with different concentrations of primers (0.2–0.8 μM) and probes (0.1–0.5 μM) for *L. martiniquensis* (**A**, **B**) and *L. orientalis* (**C**, **D**). Primer and probe concentrations and their corresponding Ct-values are listed alongside each plot. *Abbreviation*: NTC, no template control.

**Fig. 4:**
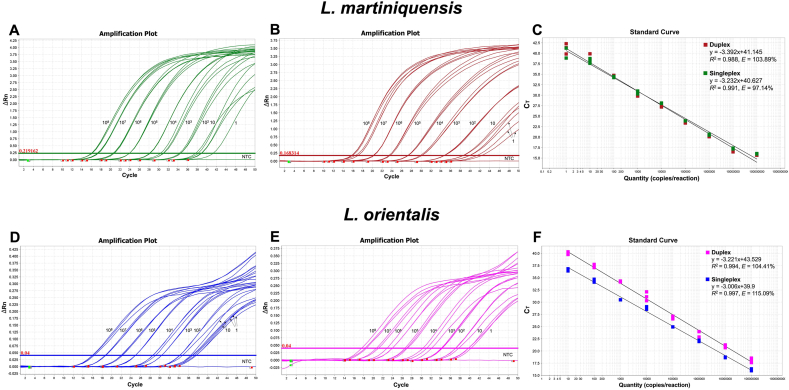
Amplification plots and standard curves of singleplex and duplex qPCR assays with 10-fold serially diluted standard plasmid concentrations of *L. martiniquensis* (**A**, **B**) and *L. orientalis* (**D**, **E**). The duplex assay was performed separately for each species, and all reactions were spiked with 10^5^ copies of the standard plasmid of the counterpart species. The standard curve plots (**C**, **F**) show the good linearity of the Ct-values for the singleplex and duplex assays. The calculated correlation coefficients (*R*^2^) and amplification efficiency (*E*) of the singleplex and duplex assays for each species are given in the graphs. *Abbreviation*: NTC, no template control.

The duplex assay was optimized using different concentrations of primers and probes. We found that the amplification performance based on delta Rn (ΔRn) and Ct-values was optimal when primer concentrations were between 0.6 and 0.8 μM and probe concentrations were between 0.3 and 0.5 μM as demonstrated in Fig. 3. Therefore, 0.6 μM and 0.3 μM were selected as the optimized primer and probe concentrations for further analysis. The amplification efficiencies of the singleplex and duplex assays for each *Leishmania* species were compared using standard plasmid templates with varying amounts of target, ranging from 10^8^ copies to 1 copy per reaction. The Ct-values of each dilution were analyzed using linear regression analysis to plot standard curves, which showed good linearity for both the singleplex and duplex assays as shown in Fig. 4. The *R*^2^-values of the singleplex and duplex assays for *L. martiniquensis* were 0.991 and 0.988, whereas amplification efficiencies were 97.14% and 103.89%, respectively. For *L.* *orientalis*, the *R*^2^-values of the singleplex and duplex assays were 0.997 and 0.994 and the amplification efficiencies were 115.09% and 104.41%, respectively.

### 3.3 Analytical sensitivity and specificity of duplex qPCR

The analytical sensitivity of the singleplex and duplex qPCR was determined based on the LOD, which represents the ability of the assay to detect the lowest concentrations of a target for each species of *Leishmania* in a tested sample. The LOD of the singleplex and duplex assays for *L. martiniquensis* and *L. orientalis* was similar at approximately one copy per reaction, as shown in Fig. 4.

The analytical specificity of the developed assay was verified by testing with *L. martiniquensis* and *L. orientalis* DNA-free samples extracted from healthy human blood, malaria blood, *Histoplasma*-infected tissue, and cultures of other *Leishmania* spp., *Trypanosoma* and *Crithidia* species. No amplification was observed for these DNA samples, indicating the high analytical specificity of the duplex qPCR developed here. However, analysis of the duplex qPCR with more diverse pathogen species will be useful to further confirm its high analytical specificity.

### 3.4 Diagnostic sensitivity and specificity of duplex qPCR

Diagnostic sensitivity and specificity were analyzed by comparing the results of the duplex qPCR with those of ITS1-PCR coupled with plasmid sequencing as a reference assay. For *L.* *martiniquensis*, 25 true positive samples were identified by both diagnostic methods, and no false negatives were found. Similarly, *L. orientalis* was detected in 5 samples by the duplex qPCR and was consistent with the ITS1 sequencing results. Therefore, these results indicate that the duplex qPCR has a diagnostic sensitivity of 100% for these two *Leishmania* species.

For diagnostic specificity, both the duplex qPCR and ITS1-PCR with plasmid sequencing were negative for *L. martiniquensis* and *L. orientalis* in 21 and 41 samples, respectively, and no false positives were found for these two species, indicating that the diagnostic specificity of this assay for *L. martiniquensis* and *L. orientalis* was 100%. In addition, the duplex qPCR showed positive and negative predictive values of 100% and perfect agreement (kappa = 1.0) with the reference assay for both *Leishmania* species as shown in Table 3.

**Table 3:** Diagnostic performance of the duplex qPCR for the detection and identification of *L. martiniquensis* and *L. orientalis* compared to ITS1-PCR and plasmid sequencing.

Species	Duplex qPCR	ITS1-PCR and plasmid sequencing		Total	
		Positive	Negative		
*L. martiniquensis*	Positive	25	0	25	PPV = 100%
	Negative	0	21	21	NPV = 100%
	Total	25	21	46	Kappa = 1.0
		Diagnostic sensitivity = 100%	Diagnostic specificity = 100%		
*L. orientalis*	Positive	5	0	5	PPV = 100%
	Negative	0	41	41	NPV = 100%
	Total	5	41	46	Kappa = 1.0
		Diagnostic sensitivity = 100%	Diagnostic specificity = 100%		

*Abbreviations*: PPV, positive predictive value; NPV, negative predictive value.

## 4 Discussion

In recent years, autochthonous cases of leishmaniasis have been increasingly reported in several countries in Southeast Asia, including Thailand (Leelayoova et al., 2017; Anugulruengkitt et al., 2022; Srivarasat et al., 2022), Myanmar (Noppakun et al., 2014), Vietnam (Tien, 2018; Vu et al., 2021), and Cambodia (Lyvannak et al., 2022). The increasing incidence of symptomatic patients has highlighted the importance of early detection, accurate diagnosis, and prompt treatment, which would lead to better treatment outcomes and prevention of disease progression and complications, especially in transmission areas. As previously described, Thailand is now considered the most endemic area for emerging *L. martiniquensis* and *L. orientalis* (Leelayoova et al., 2017; Sarasombath, 2018). Of note, *L. martiniquensis* usually presents with severe visceral leishmaniasis, which is fatal if untreated, and disseminated cutaneous leishmaniasis, particularly in immunosuppressed patients (Srivarasat et al., 2022), whereas *L. orientalis* mainly causes localized cutaneous leishmaniasis (Jariyapan et al., 2018; Anugulruengkitt et al., 2022). In addition, the drug of choice and treatment regimens for these two species are different (Mathison and Bradley, 2023). Therefore, laboratories in primary healthcare centers in endemic areas need promising diagnostic methods to confirm the causative species and detect co-infections, which would help clinicians to accurately diagnose and treat this emerging disease with precise management decisions.

In this study, we developed a duplex TaqMan probe-based qPCR assay for detecting *L. martiniquensis* and *L. orientalis* in different sample types with high diagnostic performance. The primers and probes were designed based on multiple sequence alignments of the ITS1 region and the *HSP70-I* intergenic region obtained from the GenBank database. Among the most common targets, two regions of rDNA, namely 18S rRNA and ITS1, have been used previously due to the presence of tens to hundreds of rDNA repeat units per cell, which ensures sufficient sensitivity in detection (Schönian et al., 2011; Van der Auwera and Dujardin, 2015; Galluzzi et al., 2018). The 18S rRNA region has been commonly used for detecting *Leishmania* spp. at the genus level due to its high sequence conservation (Bossolasco et al., 2003; van der Meide et al., 2008; Galluzzi et al., 2018). In contrast, the ITS1 region, which is more variable, has been previously used for detection and species identification (Talmi-Frank et al., 2010; Schönian et al., 2011; Hernández et al., 2014; Hitakarun et al., 2014), as well as characterization of intraspecific genetic diversity and phylogeographic distribution patterns of several *Leishmania* spp., including the *L. donovani*/*L. infantum* complex (Rezaei et al., 2020; Chen et al., 2021), *L. major* (Spotin et al., 2023), *L. tropica* (Charyyeva et al., 2021), as well as *L. martiniquensis* and *L. orientalis* (Ruang-Areerate et al., 2023; Ampol et al., 2024). Among the *Leishmania* ITS1 sequences analyzed in this study, a 19-bp sequence was found to be exclusive to those of *L. martiniquensis*, and this unique sequence was therefore used as the stem part of the 21-bp *L. martiniquensis*-specific probe in this developed assay.

Apart from ITS1, the *HSP70-I* gene has been extensively used as a molecular marker for PCR-RFLP and sequence analysis for *Leishmania* species differentiation and phylogenetic studies (Garcia et al., 2004; da Silva et al., 2010; Fraga et al., 2010; Hoyos et al., 2022). However, the coding regions of the *HSP70-I* gene are well conserved among closely related *Leishmania* species (Folgueira and Requena, 2007). It has previously been shown that the 3′-untranslated region (UTR) of the *Leishmania HSP70-I* gene has better discriminatory power for species typing than the coding region (Requena et al., 2012). This finding could be explained by the fact that the non-coding regions of genes appear to be under less evolutionary constraint, resulting in more sequence variability with higher discriminatory power than the coding regions (Requena et al., 2012). Therefore, we speculated that sequence variability in the intercoding region of the *HSP70-I*, consisting of the 3′-UTR, intergenic region, and 5′-UTR, would be sufficient for species differentiation across *Leishmania* spp. Then, we performed multiple sequence alignments of the intercoding region of the *HSP70-I* using the genome data from several *Leishmania* spp., revealing that the intergenic region also has a high degree of sequence variability and can be a good target for the design of primers and a probe to detect *L. orientalis* and differentiate it from *L. martiniquensis* in this study. However, we found a high sequence similarity between *L. orientalis* and the closely related *L. chancei* in the sequence region where primers and a probe were designed. Therefore, it is noteworthy that our LoHSP70IR primers and probe might theoretically amplify *L. chancei* which has only been reported from Ghana, West Africa (Kwakye-Nuako et al., 2015, 2023) and has never been reported in Thailand since its discovery.

The efficiency and *R*^2^ of *L. martiniquensis* and *L. orientalis* amplification were evaluated according to the MIQE guidelines (Bustin et al., 2009). Assay performance was determined by comparing duplex qPCR results with those of the singleplex assay. It was found that the performance of the singleplex and duplex qPCR for *L. martiniquensis* was almost similar. For L. *orientalis*, the amplification efficiency and *R*^2^ of the singleplex assay (*R*^2^ = 0.997, *E* = 115.09%) were better than those of the duplex assay (*R*^2^ = 0.994, *E* = 104.41%), possibly due to competition between primer sets during duplex amplification. In this study, all *R*^2^-values were greater than 0.98, and all *E*-values were within the acceptable range (80–120%), indicating good linearity of the standard curves and efficient amplification performance of this developed assay (Bustin et al., 2009; Kralik and Ricchi, 2017; Ruijter et al., 2021).

In addition, the sensitivity and specificity of the duplex qPCR were evaluated analytically and diagnostically. Analytical sensitivity was determined based on the LOD, which was one target copy per reaction for both *L. martiniquensis* and *L. orientalis*. Of note, both ITS1 and *HSP70-I* IR are multicopy markers (Quijada et al., 1997; Folgueira et al., 2007; Van der Auwera and Dujardin, 2015), resulting in a high analytical sensitivity of the assay. This will be clinically advantageous for detecting *Leishmania* spp. in the early stages of infection with a low parasite burden or for therapeutic monitoring, thus reducing the likelihood of false negative results (Saah and Hoover, 1997; Leal et al., 2014; Oh et al., 2016). Due to the multicopy nature of these loci, one copy does not represent a single parasite. Therefore, standard curves generated by the amplification of *Leishmania* DNA from different numbers of parasites are further required for accurate quantification of parasite load. However, the copy number of target sequences may vary between isolates or between parasite stages, probably affecting the quantitative accuracy of the assay. Alternatively, single-copy gene qPCR assays, such as the single-copy DNA polymerase I gene, which do not have these pitfalls but may be less sensitive, are recommended to confirm the infection levels (Galluzzi et al., 2018).

The duplex qPCR was tested with other non-*Leishmania* DNA samples available in our laboratory and showed no cross-amplification, indicating a high analytical specificity. Thus, the high specificity of the primers and probes developed in this study will provide us with reliable results for specific pathogen detection even in samples contaminated with DNA from other pathogen species. However, additional DNA samples from different pathogen species were recommended to confirm the high analytical specificity of the assay (Kim et al., 2024).

By evaluating different sample types, the diagnostic performance of the duplex qPCR for detecting *L. martiniquensis* was similar to that of the duplex qPCR for detecting *L. orientalis*, with diagnostic sensitivity and specificity of 100% and positive and negative predictive values of 100%. This was in perfect agreement with the standard species identification assay, i.e. ITS1-PCR and plasmid sequencing. Importantly, these results demonstrated that the duplex qPCR developed in this study is a promising tool with excellent diagnostic performance that can provide accurate species diagnosis and reduce analysis time, compared to genus-specific 18S rRNA-qPCR and ITS1 sequencing-based assay. Furthermore, we demonstrated the applicability of this duplex qPCR to screen for *L. martiniquensis* and *L. orientalis* in field-caught *Culicoides* samples, suggesting the utility of this assay for entomological surveillance to study the infection prevalence of these two *Leishmania* species in large numbers of insect samples.

Given the continuing increase in new leishmaniasis cases in endemic areas, rapid detection and accurate identification of these two autochthonous *Leishmania* (*Mundinia*) species is urgently needed for early diagnosis, prompt treatment, and epidemiological surveillance. Essentially, the TaqMan-based duplex qPCR assay was developed and validated, for the first time, for the simultaneous detection and identification of *L. martiniquensis* and *L. orientalis* in different types of biological samples with high amplification efficiency, precision, and cost-effectiveness. The novel diagnostic developed in this study would facilitate clinical diagnosis with accurate and rapid identification of autochthonous *Leishmania* spp., especially in areas of endemicity where nucleotide sequencing is not routine. It would also potentially contribute to disease management and prevention strategies to effectively reduce the spread of these neglected parasites.

## 5 Conclusions

The continuing increase in autochthonous leishmaniasis cases in Thailand represents a challenging current public health problem. Despite the increasing medical importance of leishmaniasis, diagnostic capabilities for this emerging disease remain limited. Here, we have successfully developed a novel, highly effective duplex TaqMan qPCR as a valuable tool for rapid and accurate diagnosis of autochthonous leishmaniasis, which will contribute significantly to effective disease management and treatment monitoring. In addition to diagnostic applications, this assay can also be implemented in leishmaniasis surveillance for effective prevention and control of this neglected disease.

## Funding

This research was supported by a grant from the 10.13039/501100010724Health Systems Research Institute (HSRI), Thailand (Grant No. HSRI 67-118).

## Ethical approval

The procedures of specimen collection and research methodology were reviewed and approved by the Institutional Review Board of Hatyai Hospital (HYH EC 077-65-01) and the International Review Board of the Faculty of Medicine, Chulalongkorn University, Bangkok (IRB No. 0286/67, COA No. 0757/2024) and the Animal Research Ethics Committee of the Chulalongkorn University Animal Care and Use Protocol (CU-ACUP), Faculty of Medicine, Chulalongkorn University, Bangkok, Thailand (COA No. 004/2564).

## CRediT authorship contribution statement

**Kanok Preativatanyou:** Conceptualization, Data curation, Formal analysis, Funding acquisition, Investigation, Methodology, Project administration, Resources, Supervision, Validation, Visualization, Writing – original draft, Writing – review & editing. **Nopporn Songumpai:** Formal analysis, Investigation, Methodology, Resources. **Pathamet Khositharattanakool:** Formal analysis, Investigation, Methodology. **Rinnara Ampol:** Formal analysis, Investigation, Methodology. **Chulaluk Promrangsee:** Formal analysis, Investigation, Methodology. **Chatchapon Sricharoensuk:** Formal analysis, Investigation, Validation. **Kobpat Phadungsaksawasdi:** Investigation, Validation. **Thanapat Pataradool:** Investigation, Validation. **Tomas Becvar:** Investigation, Methodology. **Barbora Vojtkova:** Investigation, Methodology. **Petr Volf:** Investigation, Methodology, Resources. **Padet Siriyasatien:** Formal analysis, Funding acquisition, Investigation, Resources, Supervision, Validation.

## Declaration of competing interests

The authors declare that they have no known competing financial interests or personal relationships that could have appeared to influence the work reported in this paper.

## Data Availability

The data supporting the conclusions of this article are included within the article. Raw data will be made available on request.
