# Supplementary material for: Novel duplex TaqMan-based quantitative PCR for rapid and accurate diagnosis of Leishmania (Mundinia) martiniquensis and Leishmania (Mundinia) orientalis, responsible for autochthonous leishmaniasis in Thailand
Source: Curr Res Parasitol Vector Borne Dis. 2024 Sep 24;6:100217. doi: 10.1016/j.crpvbd.2024.100217 (PMC11619792; doi:10.1016/j.crpvbd.2024.100217)
Supplement: Multimedia component 1 [file mmc1.docx]

**Supplementary data**

**Supplementary Table S1.** Data for *Leishmania* ITS1 sequences from the GenBank database used for the design of the primers and probe in the present study.

| **Accession no.** | ***Leishmania* species** |
| --- | --- |
| HG512946.1 | *Leishmania aethiopica* MHOM/ET/70/L96 (LEM3351) |
| FN252411.1 | *Leishmania aethiopica* MHOM/ER/2009/7457 |
| HG512964.1 | *Leishmania amazonensis* MHOM/BR/73/M2269 |
| HG512933.1 | *Leishmania amazonensis* MHOM/CO/82/CELIS (LEM2247) |
| HG512966.1 | *Leishmania braziliensis* MHOM/PE/03/LH2511 |
| FN398338.1 | *Leishmania braziliensis* MHOM/BR/00/LTB300 |
| JQ397604.1 | *Leishmania braziliensis* clone LETI-Za |
| KP006688.1 | *Leishmania chancei* MHOM/GH/2012/GH5 |
| KP006689.1 | *Leishmania chancei* MHOM/GH/2012/GH10 |
| FN687759.1 | *Leishmania donovani* MHOM/ET/2010/DM-607 |
| HG512957.1 | *Leishmania donovani* MHOM/SD/87/UGX-MARROW (LEM4272) |
| HG512907.1 | *Leishmania donovani* MHOM/IN/00/DEVI (LEM0138) |
| KM677932.1 | *Leishmania enriettii* MCAV/BR/1945/LV90 |
| HG512948.1 | *Leishmania gerbilli* MRHO/SU/87/E-11 (LEM3390) |
| HG512961.1 | *Leishmania guyanensis* MHOM/GF/2004/LBC43 (LEM4769) |
| HG512935.1 | *Leishmania guyanensis* MHOM/EC/90/UI.031 (LEM2318) |
| FR675940.1 | *Leishmania infantum* MHOM/US/10/SLRHCL1 |
| AY495830.1 | *Leishmania macropodum* AM-2004 |
| HG512963.1 | *Leishmania major* MHOM/BF/2004/REN04-8 (LEM4886) |
| FN677357.1 | *Leishmania major* MHOM/UZ/02/17h |
| KM677931.1 | *Leishmania martiniquensis* MHOM/MQ/1992/MAR1 from a patient in Martinique |
| JX195639.1 | *Leishmania martiniquensis* MHOM/TH/2011/CU1 from a Thai patient |
| OP698050.1 | *Leishmania martiniquensis* MHOM/TH/2021/CULE3 from a Thai patient |
| OM688240.1 | *Leishmania martiniquensis* MHOM/TH/2022/CULE6 from a Thai patient |

**Supplementary Table S1.** Data for *Leishmania* ITS1 sequences from the GenBank database used for the design of the primers and probe in the present study. (Continued)

| **Accession no.** | ***Leishmania* species** |
| --- | --- |
| JQ617283.1 | *Leishmania martiniquensis* Ec11010 from Florida horse |
| JX195637.1 | *Leishmania martiniquensis* MHOM/TH/2010/PCM4 strain PG from a Thai patient |
| GQ226034.1 | *Leishmania martiniquensis* from a Thai patient |
| JQ866907.1 | *Leishmania martiniquensis* clone LE-San1 from a Thai *Sergentomyia* sand fly |
| JQ001751.1 | *Leishmania martiniquensis* clone LEBM-So from a Thai patient |
| KU680857.1 | *Leishmania mexicana* MNYC/BZ/62/M379 |
| JX195640.1 | *Leishmania orientalis* MHOM/TH/2010/PCM2 |
| ON303842.1 | *Leishmania orientalis* MHOM/TH/2021/CULE5 |
| MG731230.1 | *Leishmania orientalis* MHOM/TH/2014/LSCM4 |
| MH807724.1 | *Leishmania orientalis* TR206 |
| HG512959.1 | *Leishmania panamensis* MHOM/CR/2004/TIM13 (LEM4743) |
| HG512902.1 | *Leishmania peruviana* MHOM/PE/89/LH741 |
| FN398339.1 | *Leishmania peruviana* MHOM/PE/1990/HB86 |
| KU680858.1 | *Leishmania tarentolae* p10 |
| HG512927.1 | *Leishmania tropica* MHOM/EG/90/LPN65 (LEM2001) |
| HG512925.1 | *Leishmania tropica* ISER/MA/89/LEM1694 |
| HG512949.1 | *Leishmania turanica* MRHO/SU/95/T-9551R (LEM3414) |

**Supplementary Table S2.** Data for *Leishmania* *HSP70-I* intergenic sequences from the GenBank database used for the design of the primers and probe in the present study.

| **GenBank assembly** | **Accession no.** | ***Leishmania* species** |
| --- | --- | --- |
| GCA_000444285.2 | CM007241 | *Leishmania aethiopica* L147, chromosome 28 |
| GCA_030937385.1 | CM061638 | *Leishmania amazonensis* LV79, chromosome 28 |
| GCA_024505685.1 | CM044736 | *Leishmania braziliensis* BA788, chromosome 28 |
| GCA_014466975.1 | CP048192 | *Leishmania chagasi* MCER/BR/1981/M6445/Salvaterra isolate M6445, chromosome 28 |
| GCA_017918215.1 | CM030495 | *Leishmania chancei* LV757 strain GH5, chromosome 28 |
| GCA_002243465.1 | CP022643 | *Leishmania donovani* Pasteur, chromosome 28 |
| GCA_017916305.1 | CM030603 | *Leishmania enrietii* CUR178, chromosome 28 |
| GCA_022701395.1 | CM040372 | *Leishmania guyanensis* MHOM/BR/75/M4147, chromosome 28 |
| GCA_003020905.1 | CP027827 | *Leishmania infantum* TR01, chromosome 28 |
| GCA_040363675.1 | CM080632 | *Leishmania lindenbergi* MHOM/BR/1966/M15733, chromosome 28 |
| GCA_916722125.1 | OU755562 | *Leishmania major* Friedlin, chromosome 28 |
| GCA_017916325.1 | CM030423 | *Leishmania martiniquensis* LSCM1, chromosome 28 |
| GCA_000234665.4 | FR799581 | *Leishmania mexicana* MHOM/GT/2001/U1103, chromosome 28 |
| GCA_017916335.1 | CM030459 | *Leishmania orientalis* LSCM4, chromosome 28 |
| GCA_000755165.1 | CP009397 | *Leishmania panamensis* MHOM/PA/94/PSC-1, chromosome 28 |
| GCA_040363515.1 | CM080426 | *Leishmania shawi* MCEB/BR/1984/M8408, chromosome 28 |
| GCA_033953505.1 | CP119852 | *Leishmania tarentolae* M2, chromosome 28 |
| GCA_014139745.1 | CM024314 | *Leishmania tropica* CDC216-162, chromosome 28 |
| GCA_040363505.1 | CM080391 | *Leishmania utingensis* ITUB/BR/1977/M4964, chromosome 28 |
